# A cell based, high throughput assay for quantitative analysis of Hedgehog pathway activation using a Smoothened activation sensor

**DOI:** 10.1038/s41598-017-14767-1

**Published:** 2017-10-30

**Authors:** Evgenii A. Albert, Christian Bökel

**Affiliations:** Center for Regenerative Therapies Dresden, Technical University Dresden, Fetscherstr. 105, 01307 Dresden, Germany

## Abstract

The Hedgehog (Hh) signalling cascade plays an important role in development and disease. In the absence of Hh ligand, activity of the key signal transducer Smoothened (Smo) is downregulated by the Hh receptor Patched (Ptc). However, the mechanisms underlying this inhibition, and especially its release upon ligand stimulation, are still poorly understood, in part because tools for following Smo activation at the subcellular level were long lacking. To address this deficit we have developed a high throughput cell culture assay based on a fluorescent sensor for *Drosophila* Smo activation. We have screened a small molecule inhibitor library, and observed increased Smo sensor fluorescence with compounds aimed at two major target groups, the MAPK signalling cascade and polo and aurora kinases. Biochemical validation for selected inhibitors (dobrafenib, tak-733, volasertib) confirmed the screen results and revealed differences in the mode of Smo activation. Furthermore, monitoring Smo activation at the single cell level indicated that individual cells exhibit different threshold responses to Hh stimulation, which may be mechanistically relevant for the formation of graded Hh responses. Together, these results thus provide proof of principle that our assay may become a valuable tool for dissecting the cell biological basis of Hh pathway activation.

## Introduction

Hedgehog (Hh) signalling plays an important role in development and disease, and is highly conserved across different branches of the evolutionary tree. A unique feature of the Hh signalling cascade is the sequential use of two receptor-like proteins, the actual Hh binding receptor Patched (Ptc) and the downstream, GPCR-like signal transducer Smoothened (Smo). In the absence of Hh, Ptc suppresses the activity of Smo, retaining it in an endosomal compartment. Upon Hh binding to Ptc, this suppression is released, leading to Smo translocation to plasma membrane and activation of the downstream signalling cascade. However, while the downstream events in Hh signal transduction are reasonably well understoood, the mechanisms underlying the Ptc-mediated suppression of Smo activity, and the upstream events leading to Smo activation during pathway activation, remain to be fully elucidated despite almost 30 years of research into the Hh pathway^1^.

Since Ptc is structurally a member of the RND family of small molecule transporters^2^, it has been suggested to act as a transporter for small molecules that influence Smo activity^3^. While in vertebrates attention focussed on sterol derivatives^4–6^ in *Drosophila* endocannabinoids were favoured as potential Smo ligands that may act as suppressors of Smo activity^7^ and may thus coordinate Hh signalling at the cellular and organismic level. However, it is not clear whether these endocannabinoids are the true, primary targets of Ptc activity. Instead, phospholipids represent a third class of small molecules suggested to affect Smo activity downstream of *Drosophila* Ptc. Loss of Ptc causes an increase in PI4P levels, which could be shown to promote Hh signalling^8^. More recent data provided evidence for the direct regulation of phospholipids by Hh and binding of PI4P to Smo^9^. Nevertheless, none of these molecule classes are generally accepted to constitute the major, Ptc dependent Smo regulators.

A similar research effort was focused on describing the molecular events occurring at the level of Smo during pathways activation. Most prominently, phosphorylation of *Drosophila* Smo by PKA primes it for further phosphorylation by the CK and GPRK kinases^10,11^. Both phosphorylation^12,13^ and sumoylation^14^ protect Smo from ubiquitination by interfering with ubiquitin ligases and through the recruitment of deubiquitinating enzyme, thus stabilizing Smo at the plasma membrane. Since Smo has to be present at the plasma membrane in order to activate downstream pathway components, endocytosis plays an important role in Hh pathway regulation. Indeed, trapping Smo on the plasma membrane is sufficient to promote Smo phosphorylation, thus placing Smo localization upstream of Smo activation^15^.

However, despite all these individual advances in the field, we are still lacking a comprehensive picture of the early events in Hh pathway activation. Unfortunately, screening specifically for upstream mechanisms affecting Smo activation has, to date, been difficult. Several general screens using transcriptional readouts have identified additional components of the Hh cascade, thus providing valuable insight in our understanding of the system^16–20^. Nevertheless, this strategy also has limitations. Most prominently it responds to the final outcome of pathway activation. It is therefore likely to miss events that partially perturb Smo activation but whose effect on gene expression may be buffered or masked by downstream components of the cascade, e.g. through signal amplification and feedback mechanisms. A system that would allow us to directly follow Smo activation, uncoupling it from internal feedback processes, would circumvent this problem, and help shedding light specifically on the upstream events of pathway activation.

**Figure 1:**
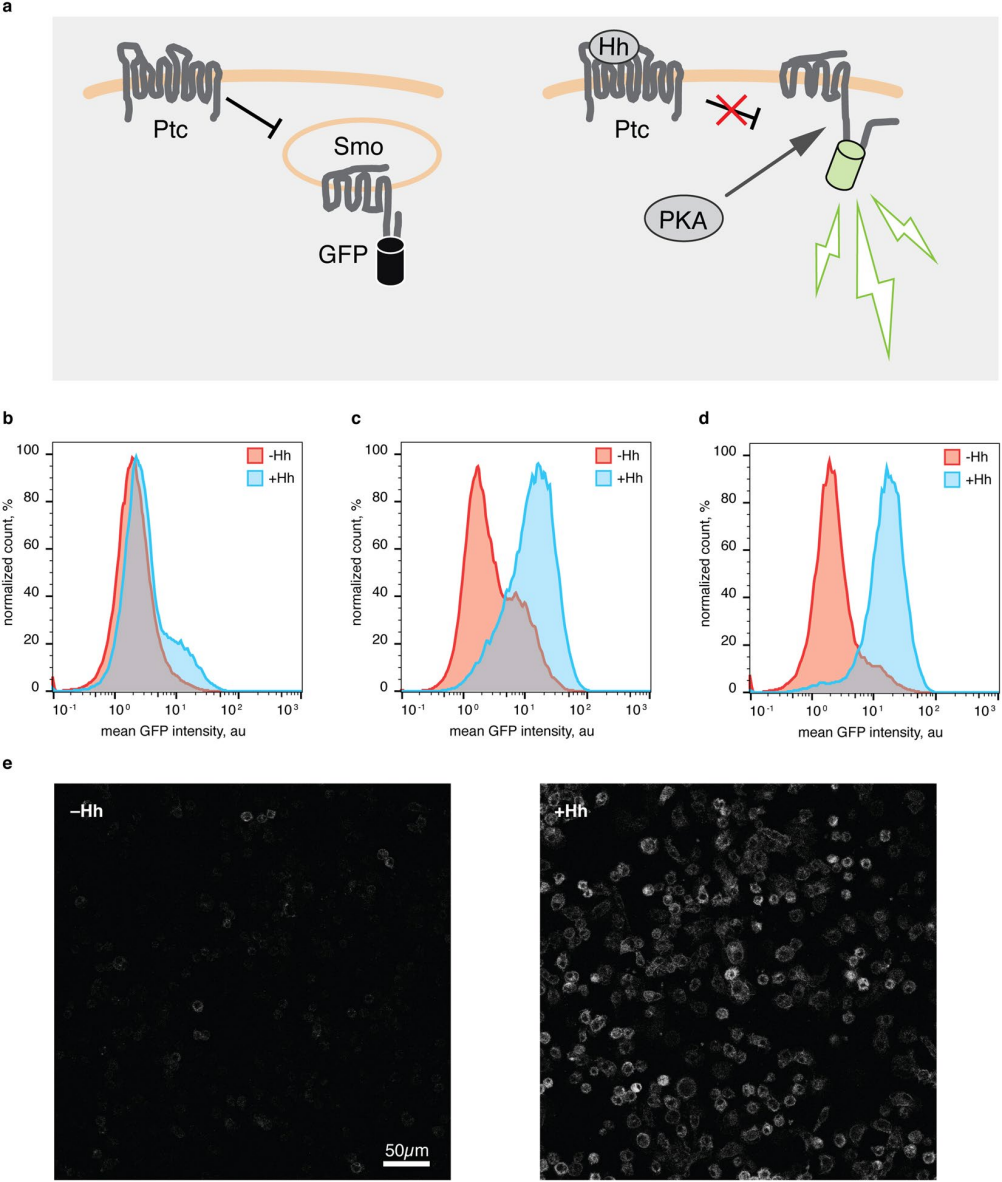
Direct detection of Smo activation using a SmoIP transgenic S2 cell line. (**a**) SmoIP detection principle. In the inactive state, an intramolecular loop in the Smo cytoplasmic tail forces an inserted cpGFP cassette from the Inverse Pericam (IP) Ca^2+^ sensor into a nonfluorescent conformation. During pathway activation, dissolution of this loop by Smo phosphorylation allows the IP cassette to relax into a fluorescent state. (**b**–**d**) FACS analysis of UAS-SmoIP transgenic cell lines following 24 h stimulation with Hh conditioned medium. Cells were screened as a polyclonal line following 3 weeks selection (**b**), following iterative sorting of strongly responding cells (**c**), and as a single cell derived, clonal line (cl14) (**d**). (**e**) Confocal live-cell images of cl14 response to Hh.

We have previously described a fluorescence based sensor (SmoIP) that can visualize endogenous or experimental phosphorylation of *Drosophila* Smo in transgenic flies^15^ by detecting the associated disruption of an off-state specific intramolecular loop in the Smo cytoplasmic tail^21^. For this, the circularly permutated GFP (cpGFP) core of the Inverse Pericam Ca^2+^ sensor^22^ was inserted into the C-terminal Smo cytoplasmic tail such that the formation of the intracellular loop forces the cpGFP into an nonfluorescent state, while the release of the loop by phosphorylation lets the cpGFP core relax into a fluorescent conformation. The tight correlation between the phosphorylation-induced conformational change, downstream signalling activity, and reporter fluorescence was validated through non-phosphorylatable and phosphomimic variants^15^. (Fig. 1a). Using this sensor in a screening setup could potentially overcome the limitations of the transcriptional readouts, and would allow to systematically investigate pathway activation at the receptor level.

Here, we report the development of a cell-based assay based on the SmoIP sensor for the direct, quantitative, high throughput assessment of the Smo activation state. Using this system we could first demonstrate that individual cells within a largely homogenous, clonal population activate Smo at cell specific threshold concentrations of Hh. Second, we performed a proof of principle experiment, testing a library of small molecule inhibitors with defined molecular targets for their effects on Smo activation. We thereby identified components of the MAPK pathway as well as the polo and aurora kinases involved in cell cycle regulation as two major clusters of target proteins, whose inhibition affects Smo activation through distinct molecular mechanisms. These observations thus provide proof of principle for the suitability of our assay for high throughput screening experiments, and provide novel insights in the logic of Smo activation.

## Methods

### Plasmids

The P-element vectors pUAST or pCasPer using the appropriate inserts were used for the generation of cell culture plasmids. To generate the UAS::smoIP plasmid, the white^+^ transgenesis marker was of pUAST-SmoIP was replaced with act::Hygro and tubulin::Gal4VP16 cassettes. For tubulin::smoIP, the act::DHFR selection cassette was cloned into the backbone of pCasPer4-tubulin::SmoIP in place of the white^+^ marker. For mtn::HhN, an act::Hygro selection cassette was cloned into the backbone of a pCasPer4 plasmid carrying a mtn::HhN insert. Detailed plasmid maps are available upon request.

### Cell culture

S2 cells were cultivated at room temperature in Schneider’s Drosophila Medium (Pan Biotech) supplemented with 10% FBS (ThermoFisher) and pen/strep antibiotics in T25 flasks (TPP). Cell transfection was done using a standard calcium phosphate protocol^23^. For P-element transformation, target plasmid was co-transfected with ∆2-3 helper plasmid^24^ at a 10:2 ratio. Stable cell lines were selected on either 300 µg/ml hygromycinB (Sigma-Aldrich) or methotrexate 4*10^−7^ M (Sigma-Aldrich) for 3 weeks^25^. Clonal selection was done according to protocol^26^. Briefly, S2 feeder cells were irradiated with 23.3 kR. Target cells were diluted to a concentration of 50 cells/ml, mixed with feeder cells (10*10^6^) in 8 ml of full growth medium. 2 ml of 1.5% agarose (filter sterilized) were added to the cells and mixed by gentle shaking. Individual clonal colonies were excised in 2-3 weeks after reaching 2–3 mm in diameter, the surrounding agar was mechanical softened and cells were placed in 96 well plates (ThermoFisher). Expression from the mtn promoter was induced using 1 mM of CuSO_4_. Hh condition medium was collected from cells after 7d of growth, filter-sterilized and stored at 4 °C for up to 5 months. Mock medium was produced in the same fashion by wild type S2 cells. For all experiments except screens and western blots cells were stimulated in 96 well plates at a density of 10^5^ cells/well with 20 µL of conditioned medium. Fluorescence was measured after 24 h not specified otherwise. For ds RNA experiments cells were seeded on 96 well paltes in CCM3 medium (ThermoFisher) at a density of 30*10^3^ cells in 50 µL containing 2 µg dsRNA (Sheffield iRNA screening facility) per well. Cells were analyzed 4 days later. Inhibitors were used in the following concentrations: OA, 5 nM; IBMX, 24 µg/ml, fsk, 80 µM; h89, 30 µM, dbn, tak-733 and vlt, Osi-027, MK-8669 15 µM if not stated otherwise.

Cl8 cells were cultivated under standard conditions in M3 medium (Sigma-Aldrich, USA), supplemented with 2.5% FBS (ThermoFisher, USA), and 2.5% fly extract (prepared according to the DGRC protocol), insulin at 5 µg/ml and pen/strep solution.

### FACS

Cells were analyzed either by MACSQuant (Miltenyi biotec) or by FacsCanto II analyzers (BD). Fluorescence was acquired directly in the growth medium after cells detachment by pipetting. Cell sorting was done on a facsAria II sorter (BD).

### Screening

Cl14 cells were dispensed to 384 well V-bottom plates (Eppendorf) containing a pre-aliquoted library of small molecule inhibitors (Selleckchem, Houston, USA, library L1100) at concentrations of either 2 µM or 15 µM, in 25 µl of medium at a density of 25*10^3^ cells per well. After 30 min of incubation cells were stimulated with 5 µL of Hh conditioned or mock medium. After 24 h, 4000 cells per well were analyzed using a BD cantoII FACSanalyzer.

### Ubiquitination assay

Cl14 cells were stimulated with Hh-conditioned or mock medium for 24 h 0.5 h prior to lysis 50 µM of MG132 was added to medium. SmoIP protein was extracted from cell lysates using the µMACS GFP isolation kit (Milteny Biotec, Germany) and subjected to western blot analysis.

### Western blots

3*10^6^ S2 cells were seeded on 6 well plates in 1,5 ml volume and stimulated with 500 µL of either Hedgehog or mock conditioned medium for 24 h hours. Cells were lysed in lysis buffer (25 mM Tris pH 7.2, 150 mM NaCl, 5 mM MgCl2, 0.2% NP-40, 1 mM DTT, 5% glycerol) on ice in presence of protease (Roche) and phosphatase (Sigma-Aldrich) inhibitor cocktails for 30 min. Lysates were boiled for 20 sec with loading buffer, cooled on ice an run on bis-tris gradient gels (ThermoFisher). Ptc (Apa 1, 1:250), smo (20C6, 1:100), and tub (12G10, 1:250) antibodies were obtained from DSHB, beta-act (1:5000) from Abcam, FK2 anti-ubiquitin antibodies (1:1000) from Cayman, GFP antibody was obtained from Clontech. The HhN antibody (1:500) was a gift of Suzanne Eaton (Dresden). Primary antibody incubation was performed at 4 °C overnight, secondary antibody incubation for 1 h at RT.

### Plasma membrane smo staining

After stimulation for 24 h with Hh conditioned medium 2*10^6^ cl14 cells were harvested by pipetting and centrifugation, re-suspended in 150uL FACS buffer (0.1% NaN_3_, 0.5% BSA in PEM) containing Smo ab (1:300) and stained for 1 h 30 min at RT on shaker. Then cells were centrifuged, washed once with 2 ml of ice-cold FACS buffer for 10 min, centrifuged and stained with AlexaFluro 647 tagged secondary antiserum (1:500) for 30 min at RT. Cells were then centrifuged, washed, and centrifuged again, re-suspended in 200 µL of FACS ice-cold buffer and analyzed by FACS.

### Real time qPCR

3*10^6^ S2 cells were treated with inhibitors in 2 ml medium in 35 mm petri dishes for 24 h before RNA extraction using Trizol (ThermoFisher). cDNA was synthesized using a first strand cDNA synthesis kit (ThermoFisher) and random primers from 3 µg of RNA. qPCR was performed with maxima SYBR green qPCR master mix (ThermoFisher) on a lightCycler 480 (Roche) using the following primer pairs:

ptc_F: AGTCCACGAACAATCCGCA

ptc_R: TGGGTCGTCTGAATGAGCAG

gapdh2_ F: GAGTTTTCGCCCATAGAAAGC

gapdh2_R: CGATGCGACCAAATCCATTG^27^

Ptc expression was calculated using the ddCt method relative to gapdh2 expression.

### Microscopy

Cells were live-imaged 24 h after stimulation with Hh condition medium. 2–3 h prior to imaging cells were lifted by pipeting and placed in Lab-Tek chambers (ThermoFisher). Images were taken using Zeiss LSM780 confocal microscope and analyzed by ImageJ.

### Data analysis

FACS data was analysed by FlowJo (FlowJo Software, USA). Gating for FSC and SSC axis in each experiment was done according to the control cells. For the screen results, only samples with more than 50% of cells in the chosen gate were taken in to account. Median GFP intensity was calculated for the gated population in each sample. Treatment effects were measured as a difference between signal intensity of inhibitor and a non-stimulated control normalized to the difference between stimulated and non-stimulated controls, hereafter referred to as “normalized response”. Analysis and visualization of the screen results was done with the help of the Pandas Python package. All charts represent mean ± sd values with sample sizes indicated in figure legend. Significance levels were calculated using the Mann-Whitney U-test as implemented in the ScyPy Python package.

## Results

### The cell-based SmoIP assay allows direct detection of Smo activation

To develop a system for the direct investigation of Smo activation in cell culture, we chose the *Drosophila* S2 cell line^28^. These cells express all relevant upstream components of the Hh pathway, but lack the downstream transcription factor Ci and are therefore unable to affect Smo activation through transcriptional feedback^29^. Expression of the SmoIP sensor from the endogenous promoter yielded insufficient fluorescent signal. To obtain the expression levels required for reliable detection we turned to reporter overexpression, re-establishing a corresponding polyclonal line expressing SmoIP under control of a UAS promoter driven by a tubulin-Gal4VP16 cassette on the same plasmid. We also switched to hygromycin selection, which was expected to produce a higher, average transgene copy number per cell^25^. Indeed, stimulation of S2 cells transfected with UAS-smoIP tub-Gal4VP16 (in the following abbreviated as UAS-smoIP) with Hh conditioned medium produced a fluorescent signal readily detectable by FACS. However, the proportion of transgenic cells in the population was too low for robust analyses (Fig. 1b). To increase the fraction of transgenic cells in the population we iteratively sorted and recultured the 10% of cells with highest response to Hh stimulation based on sensor fluorescence. Already after two rounds of sorting about 90% of cells were responsive (Fig. 1c). Since P-element based transgenesis produces random integration in the genome we decided to subclone individual cells to produce genetically homogeneous lines with the desired response properties for use in subsequent experiments. Using a soft agar cloning technique in combination with an irradiated feeder cell layer^26^ we obtained and analyzed 20 individual clones harboring UAS-SmoIP insertions. For the following analysis we selected clone 14 (cl14), which showed the highest ratio of induction signal over background pathway activity (Fig. 1d,e). As a result of these preparatory experiments we had therefore obtained a stable, genetically homogeneous clone of S2 cells that reproducibly responded to Hh stimulation with a robustly detectable increase in sensor fluorescence.

### UAS-SmoIP fluorescence responds as predicted to experimental manipulation

**Figure 2:**
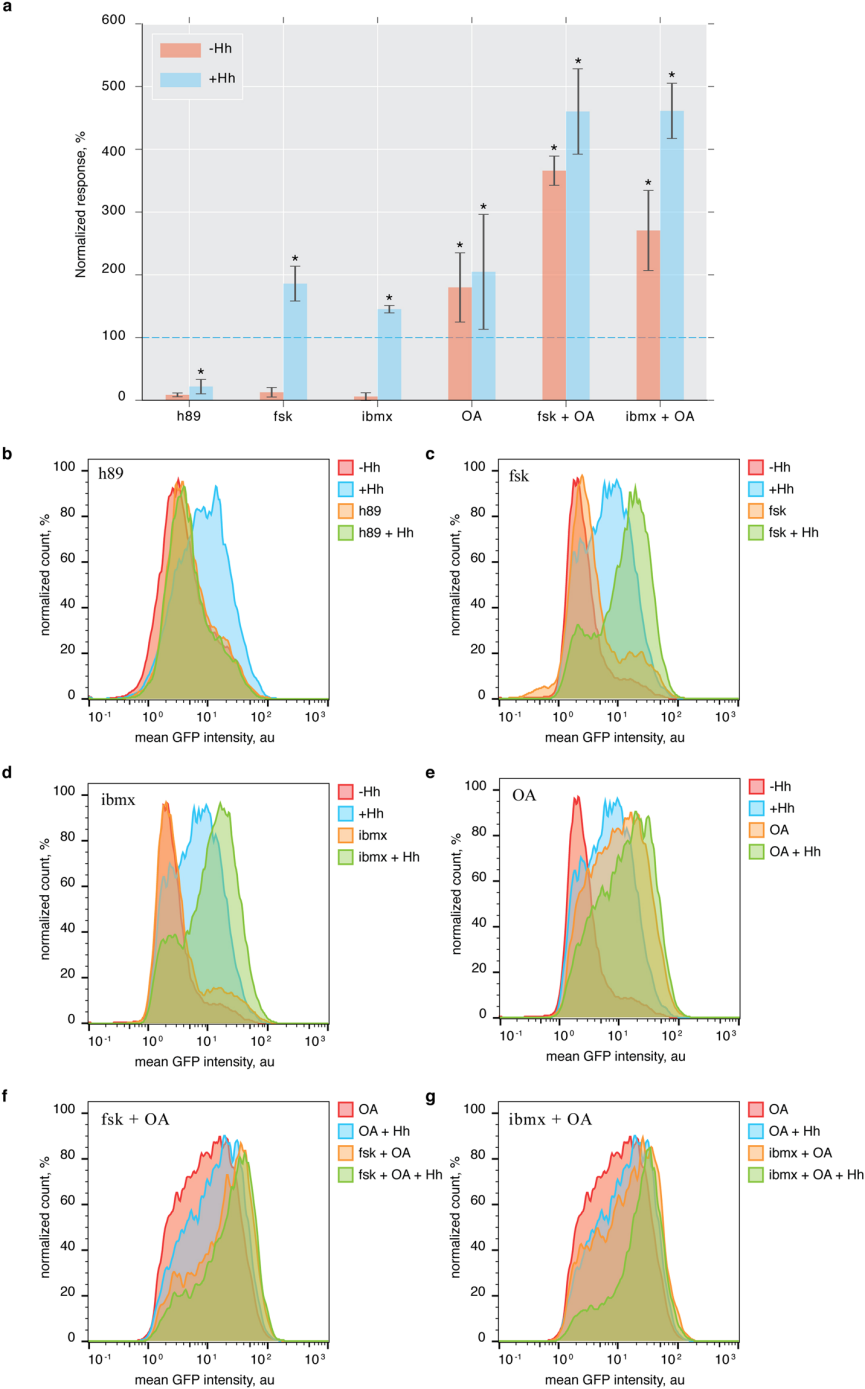
The SmoIP assay captures the endogenous pathway response to chemical perturbation. (**a**) Average effect of h89 (PKA inhibitor), fsk, IBMX (PKA activators), and OA (phosphatase inhibitor), alone or in combination, on baseline and Hh induced SmoIP fluorescence response. Effect of Hh stimulation on untreated cells set to 100% (dashed line). N = 3–6 replicates per experiment, *p < 0.05 relative to control Hh stimulation, Mann-Whitney U-test, error bars indicate SD. (**b**–**g**) FACS histograms for individual experiments using the indicated components.

To validate that the cl14 assay correctly reflects endogenous Hh pathway behavior we tested system response to perturbation of known components of the Hh signalling cascade. PKA is the main kinase that phosphorylates Smo and promotes its activation. Consistently, the small molecule PKA inhibitor h89^30^ completely abolished SmoIP fluorescence in response to Hh treatment (Fig. 2a,b). To test whether PKA activity was sufficient for the full activation of our assay we treated cells with two PKA activators, forskolin (fsk) and IBMX, that had both previously been shown to work in *Drosophila* S2 cells^31,32^. Even though both inhibitors activate PKA by increasing intracellular cAMP levels, they do so by different mechanisms: While fsk stimulates adenylate cyclase activity, IBMX inactivates phosphodiesterases. Importantly, the two PKA activators exhibited consistent effects on SmoIP sensor fluorescence: In both cases, application of the drug had barely any effect on mock treated cl14 cells, but showed pronounced, cooperative activation when acting together with Hh (Fig. 2a,c,d), reproducing previously published results for fsk^31^. In contrast, global inactivation of phosphatases by okadaic acid (OA) is sufficient to drive Hh signalling in the absence of ligand^33^. Consistently, OA treatment by itself induced a sensor response exceeding the fluorescence induced by stimulation with Hh alone (Fig. 2a,e). Interestingly, combined treatment of cl14 cells with OA and Hh did not increase UAS-smoIP response strength over OA alone. In contrast, treatment with fsk or IBMX in combination with OA had a pronounced cooperative effect on Smo activation that could not be further increased by addition of Hh. These observations suggest that, in the absence of Hh, phosphatases sensitive to OA continuously act to maintain Smo in an inactive state, and that the resulting Smo pool cannot by accessed by the endogenous PKA, even when this kinase is experimentally activated. Instead, activated PKA requires some prior action on Smo, provided endogenously by Hh or experimentally by OA, before it can promote Smo activation (Fig. 2a,f,g).

As a final test of assay specificity we knocked down several pathway regulators by bathing with dsRNA (Supplementary Fig. S1). Knockdown of Smo, which also eliminates the sensor itself, and Ptc, which mimics stimulation with Hh, were used as positive and negative controls, respectively. While Smo dsRNA reduced sensor fluorescence, Ptc, knockdown resulted in sensor activation even in the absence of ligand. Protein stabilization is known to play a major role in Hh signal transduction. Degradation of Smo via the proteasome/lysosome pathway ensures that signalling is kept down in the absence of ligand^12,13^. Accordingly, knockdown of Uba1, the sole E1 enzyme in *Drosophila*, slightly increased sensor fluorescence in the absence of Hh. Finally, inhibition of endocytosis by knockdown of the *Drosophila* Dynamin homologue Shibire, which we had previously shown to be sufficient to induce Smo activation^15^ strongly increased the sensor signal, exceeding the values achieved by stimulation with Hh alone.

Summarizing these experiments we could therefore conclude that the cl14 system correctly reports Smo activation in cell culture in response to both Hh stimulation and experimental perturbation of the signalling machinery.

### Characterising the response kinetics of the cl14 assay

The assay system we had developed and validated gave us an opportunity to determine the Smo response to Hh stimulation quantitatively and with single cell resolution. This approach promised to shed light on the how target cells may respond to graded Hh signals, a recurring and important theme in developmental biology. However, we could not assume that a fluorescence based assay in S2 cells that do not normally respond to Hh would reflect pathway activation with the same rapid kinetics reported previously for salivary glands *in vivo* ^34^ or endogenously Hh sensitive Cl8 cells *in vitro* ^35^, where increases in Smo protein level were detectable as soon as 30 min after onset of stimulation. We therefore first decided to explore how the cl14 sensor system responded over varying stimulation times.

**Figure 3:**
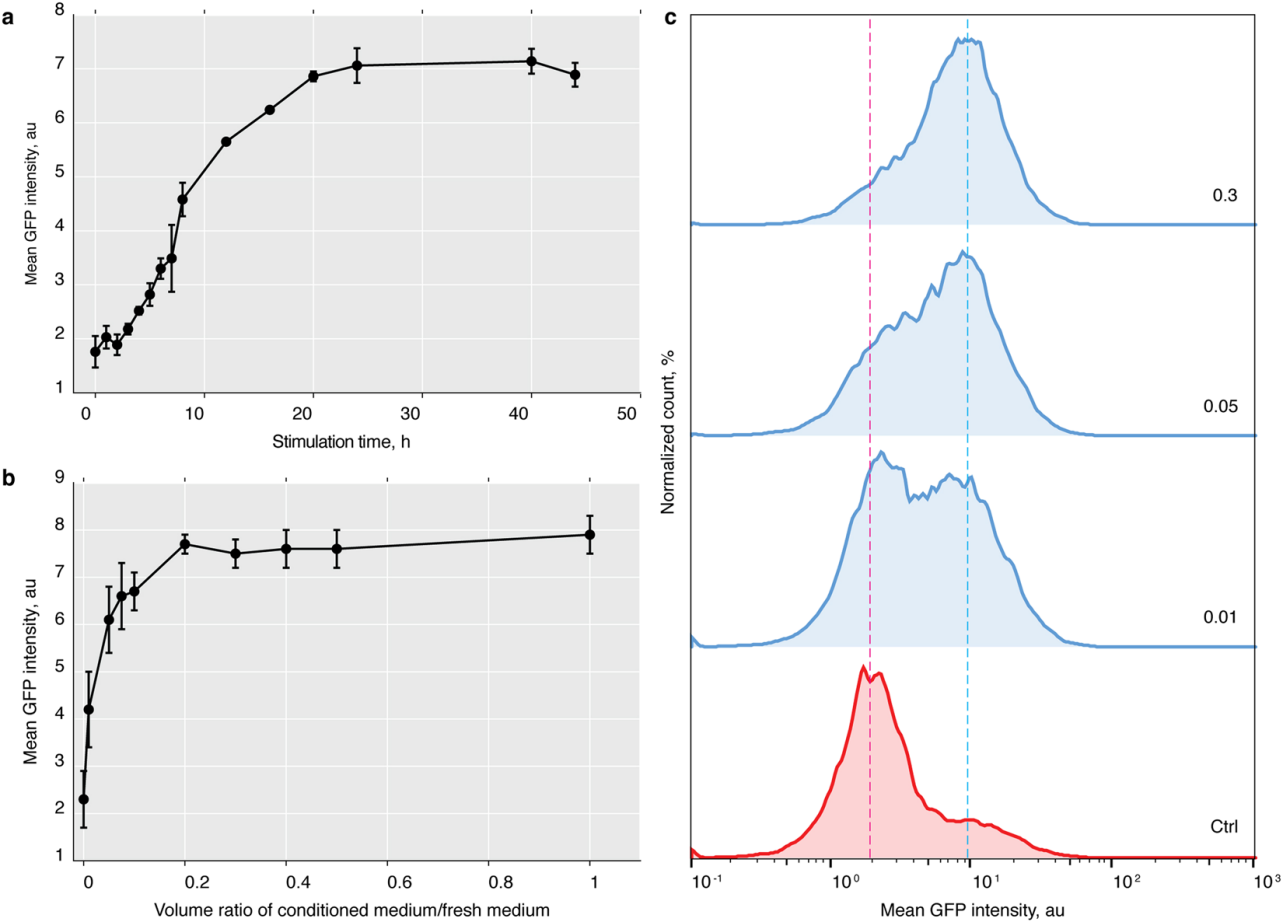
Time and concentration dependence of the SmoIP response. (**a**) SmoIP fluorescence signal of cl14 cells stimulated 1:1 with Hh conditioned medium plotted against stimulation time (**b**) SmoIP fluorescence signal of cl14 cells after 24 h of stimulation plotted against ratio of Hh conditioned and fresh medium. (**c**) FACS histograms for individual points of the concentration curve, numbers represent ration between condition and fresh medium. (**a**,**b**) N = 2–6 replicates, error bars indicate SD.

For stimulation of cultured cells a 1:1 mixture of fresh and Hh conditioned medium is typically assumed to provide full pathway activation^36^. We therefore used this mixing ratio to build the time response curve of our assay. Upon stimulation, the fluorescent sensor signal became first detectable after 4 h and then gradually increased up to 24 h under continuous stimulation (Fig. 3a). The slower kinetics of our system may reflect a lower sensitivity of the fluorescence readout compared with immunoblotting, therefore requiring prolonged signal integration and protein stabilization. To maximise the detection window of our assay we fixed stimulation time at 24 h throughout all further experiments.

### Stimulation of cl14 cells with increased Hh levels reveals cell specific response thresholds

We next explored the volume ratio between conditioned and fresh medium in a range from 1:100 to 1:1 (Fig. 3b). The system reached saturation around a ratio of 1:5. However, we were able to detect pathway activation even at the lowest concentrations tested. Taking advantage of the fact that the FACS based assay provides information about signal intensity for each individual cell we broke the average values presented in (Fig. 3b) down to response histograms for fractions of Hh conditioned medium ranging from 0 to 0.3. Interestingly, at intermediate stimulation levels the average fluorescence signals exhibited a bimodal distribution, comprising of two populations of cells with high and low reporter fluorescence. Rather than shifting the total population towards higher fluorescence levels, increasing Hh levels thus caused a larger fraction of the cells to activate their SmoIP sensor (Fig. 3c). Even though our cl14 assay line is clonally derived from a single cell, each cell in the tested population thus appears to have a different concentration threshold above which it activates its Hh signalling cascade.

### A small molecule inhibitor screen suggests an effect of RTK signalling and cell cycle associated kinases on Smo activation

The real power of *in-vitro* assays such as the one we developed lies in the possibility to perform high throughput experiments. We therefore decided to optimize our assay for future high throughput studies. This was facilitated by the fact that S2 cells can be cultivated in suspension, which allowed us to easily implement FACS as a detection system and thus immediately obtain quantitative data. To minimize screening time, a typical problem of FACS based assays, and to increase throughput, we scaled the assay down to 384 well plate format.

**Figure 4:**
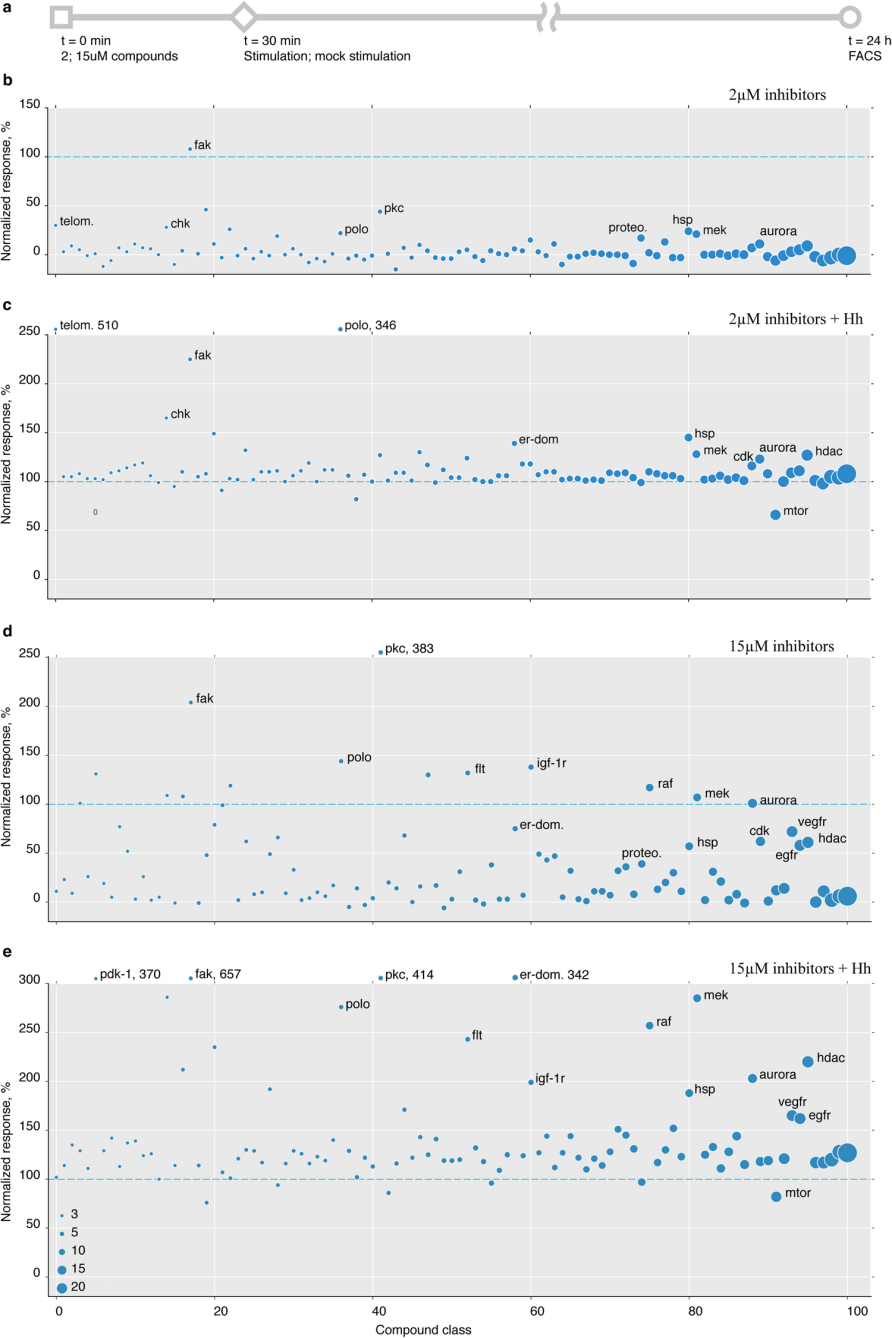
Screening a small molecule inhibitor library for modulation of SmoIP fluorescence. (**a**) Schematic representation of screen setup. (**b**–**e**) Normalized effect of inhibitors grouped according to their primary targets on SmoIP fluorescence. Effect of Hh stimulation on untreated cells set as 100% (dashed line). Diameter of circle reflects number of components per cluster, cutoff N ≥ 3, clusters sorted along X axis accordingly. (**b**,**c**), inhibitors used at 2 µM, (**d**,**e**) at 15 µM.

As proof of principle we systematically tested a Selleckchem collection of 1130 small molecule inhibitors with known molecular targets (Selleckchem L1100) for effects on Smo activation state. However, these inhibitors were developed and validated for mammalian systems, and for the majority there is no information about their efficacy in *Drosophila*. We therefore screened each compound at two concentrations, 2 µM and 15 µM. Cells were pre-incubated with compounds for 30 min, stimulated with either Hh or mock conditioned medium, and analyzed by FACS after 24 h (Fig. 4a). The effect of each given treatment was normalized to the increase in sensor fluorescence caused by Hh stimulation of otherwise untreated cells, which was set as 100%. Measuring the effects of inhibitors on stimulated and unstimulated fluorescence baselines enabled us, in principle, to detect both activators and inhibitors of Smo activation.

Surprisingly, we found few compounds capable of downregulating Smo activation upon stimulation with Hh. In contrast, many more compounds increased Smo activation, producing a continuous spectrum of responses ranging from mild increases up to 10–20 times over control (Supplementary Fig. S2a, Supplementary Table S1). We therefore clustered the compounds according to their molecular targets and computed median response for each of these classes (Fig. 4b–e, Supplementary Table S2).

Hits were then identified as clusters where multiple inhibitors targeting one or more components of the same pathway exhibited a consistent effect on sensor fluorescence. Correlating the observed effect for each such cluster between experiments performed at different concentrations and in the presence vs. absence of Hh stimulation demonstrated reproducibility and robustness of our assay (Supplementary Fig. S2b).

**Figure 5:**
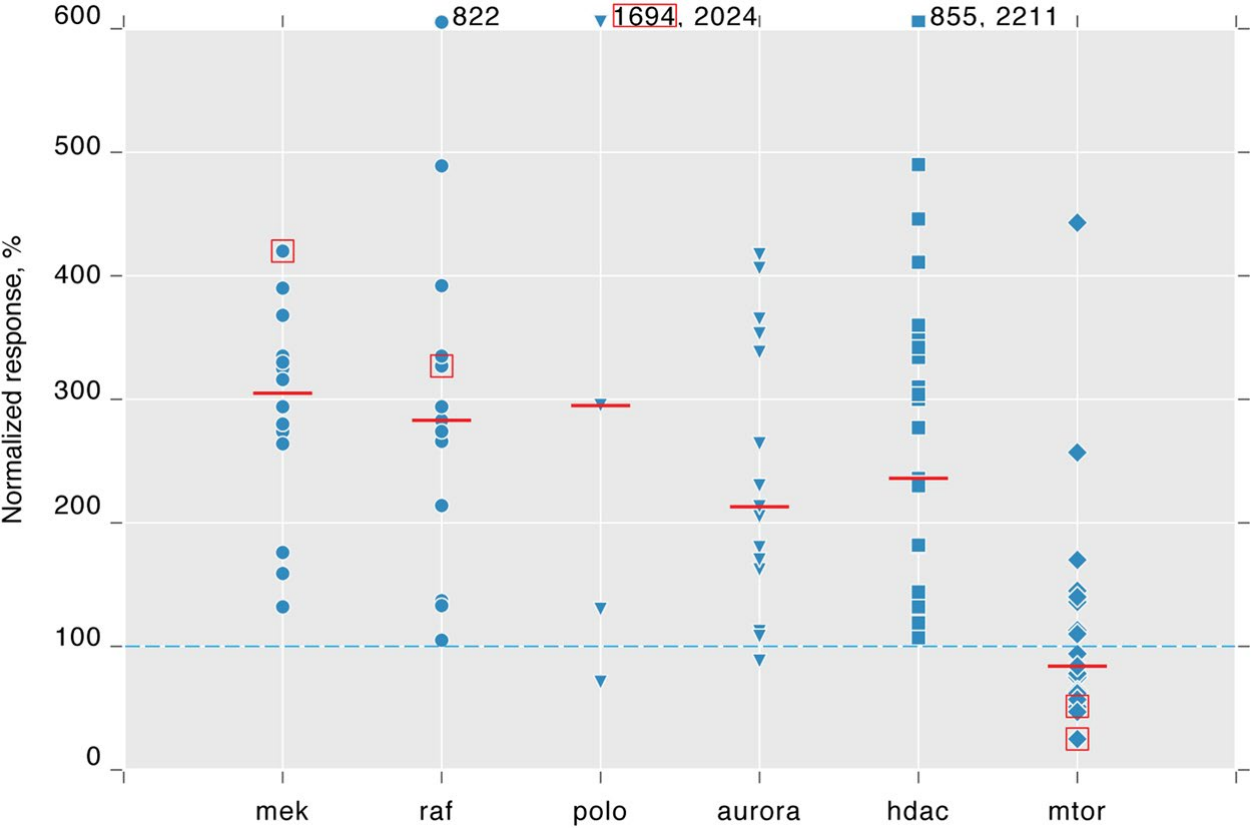
Inhibitor classes with consistent effect on Smo activation. SmoIP fluorescence of cl14 cells stimulated for 24 h with Hh conditioned medium in the presence of individual compounds inhibiting the indicated, selected primary targets. Effect of Hh stimulation on untreated cells set as 100% (dashed line). Inhibitor concentration 15 µM, red bars indicate median, red boxes mark compounds selected for subsequent independent validation experiments.

Clustering compounds by targets in this way revealed several candidate pathways whose inhibition appeared to modulate Smo activation. Sensor fluorescence was increased by compounds targeting various RTKs (Fig. 4b–e) or the downstream Raf and MAPK signalling complexes (Figs 4b–e and 5). A second cluster exhibiting an increased SmoIP signal was comprised of compounds targeting kinases regulating cell cycle progression and microtubule organization (polo-like and aurora kinases) (Figs 4b–e and 5). A third cluster of compounds increasing sensor fluorescence consisted of inhibitors of histone deacetylases (Figs 4b–e and 5). The sole class of inhibitors consistently downregulating sensor response to Hh was the group of mTOR inhibitors: More than half of these compounds (15/23) reduced the fluorescence response to Hh stimulation by at least 20% (Figs 4b–e and 5).

Thus, our screen implicated three major signalling pathways and the epigenetic machinery in the regulation of Smo activation, neither of which had previously been shown to act near the top of the Hh pathway.

### Validation of hits via independent, secondary assays confirms the links between MAPK pathway or polo kinase inhibitors and Smo activation

The mTOR protein is part of MTORC1 complex that acts as a central regulator of cell growth and protein synthesis^37^. Compounds targeting mTOR consistently reduced reporter fluorescence in response to Hh relative to control (Fig. 5). However, Smo protein is constantly turned over. Its accumulation in response to Hh, especially over the long stimulation period of our assay, thus requires *de novo* protein synthesis, which could be expected to be sensitive to mTOR inhibition. Consistently, both reporter accumulation and reporter fluorescence in response to stimulation with Hh was reduced by treatment with two different mTOR inhibitors (Supplementary Fig. S3). We therefore decided to focus instead on the compound clusters causing increased sensor fluorescence, for similar reasons excluding the cluster of HDAC inhibitors, which as epigenetic modifiers most likely affected Smo activation indirectly.

From the remaining clusters, we selected three highly scoring compounds: To interfere with signalling downstream of RTKs at two different levels we chose the Raf inhibitor dabrafenib (dbn)^38^ and the MEK inhibitor tak-733^39^. Since the two kinases act sequentially within the same signalling cascade, a shared mode of Smo activation by their respective inhibitors would lend credibility to our assay. Volasertib (vlt)^40^ was used as an example of a polo inhibitor strongly affecting Smo activation (Fig. 5).

**Figure 6:**
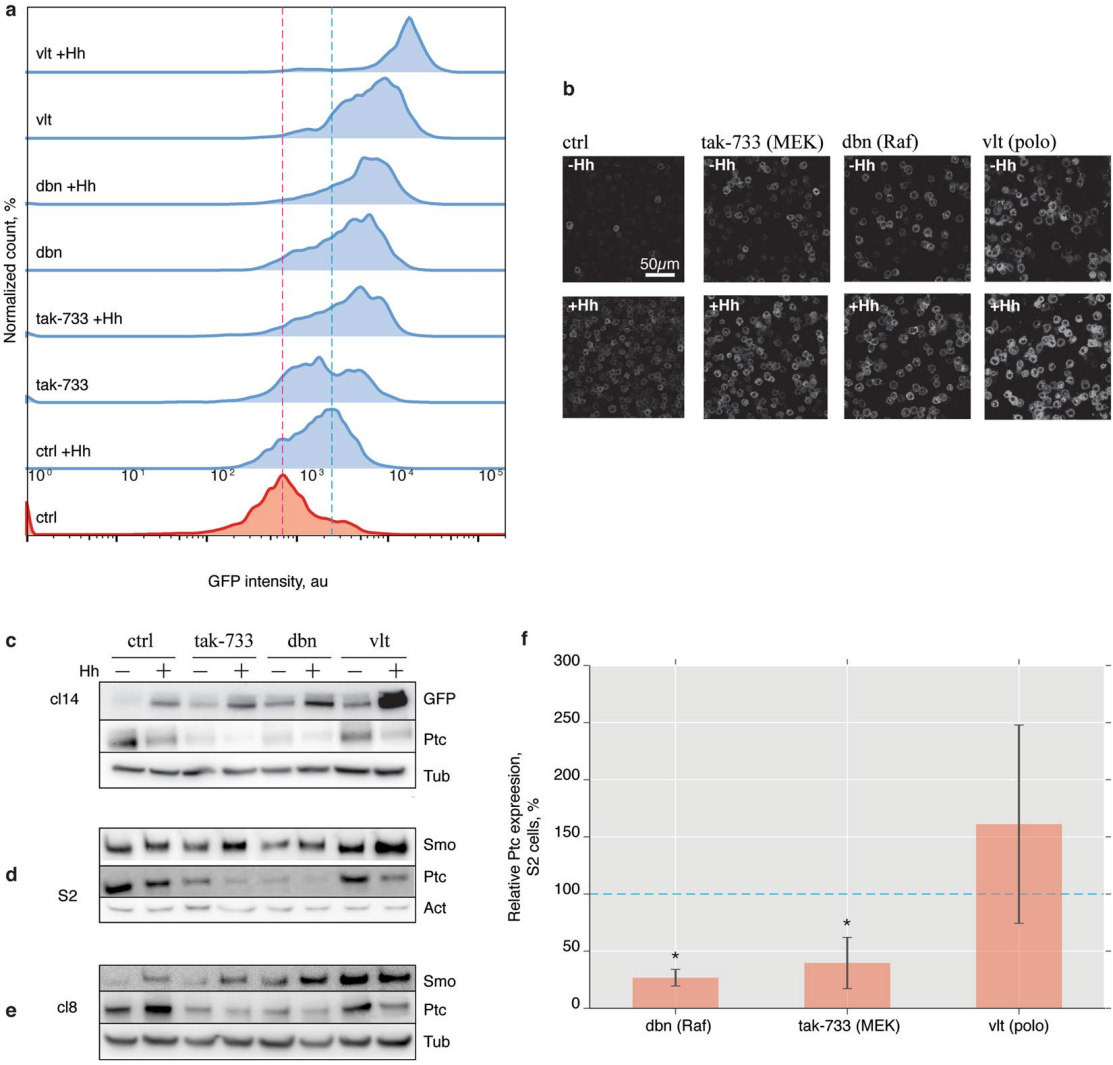
Validation of screen results for selected inhibitors. (**a**) FACS histograms of SmoIP fluorescence following treatment with 15 µM tak-733 (MEK inhibitor), dobrafenib (dbn, Raf inhibitor) or volasertib (vlt, polo inhibitor) in the presence or absence of Hh. Red and blue dashed line indicate baseline and Hh induced fluorescence, respectively, in untreated control cells. (**b**) Confocal live-cell images corresponding to the histograms in (**a**). (**c**–**e**) Western blots for GFP, Smo and Ptc on lysates of Cl14 (**c**), S2 (**d**) and cl8 cells (**e**) in the presence of indicated inhibitors. Tubulin (**c**,**e**) or actin (**d**) used as loading control. (**f**) Ptc transcription in S2 cells following inhibitor treatment. Expression normalized to GAPDH2, N = 3–4, mean ± sd, *p < 0.05, Mann-Whitney U-test.

We first confirmed that incubation of cl14 cells with either compound for 24 h did not lead to increased cell death or abnormal cell morphologies observed by changes in FACS forward and side scatter or by microscopy. The selected compounds thus do not appear to cause nonspecific cytotoxicity. Second, we repeated the FACS experiments at larger scale and tested the induction of sensor fluorescence by live cell microscopy. All three compounds stimulated fluorescence of the SmoIP sensor at levels higher than Hh treatment alone (Fig. 6a) and caused accumulation of activated fluorescent SmoIP sensor at the cell periphery (Fig. 6b). Induction of sensor fluorescence by all compounds was concentration dependent, and did not inadvertently induce Hh production by the cl14 cells (Supplementary Fig. S4), further supporting the initial observations made in screening mode and suggesting that the observed effects were specific.

However, assay conditions, including expression levels and stimulation times, were optimized to achieve the maximal fluorescence signal. Activation of the sensor did therefore not necessarily directly reflect activation of the endogenous signalling cascade. We therefore confirmed our FACS and microscopy results by methodologically independent biochemical experiments in our cl14 reporter cell line (Fig. 6c), as well as in wildtype S2 cells (Fig. 6d) and in cl8 cells (Fig. 6e), an unrelated cell line that expresses the entire Hh signalling cascade including the transcription factor Ci and is thus endogenously sensitive to Hh^29^. Stimulation with Hh conditioned medium or inhibitor treatment of cl14 cells individually already caused increased SmoIP protein levels. This response was further enhanced by combining ligand and inhibitors (Fig. 6c). A consistent effect, albeit with a reduced dynamic range, was also seen for endogenous Smo protein in S2 cells (Fig. 6d) and in cl8 cells (Fig. 6e). Thus, activation and accumulation of SmoIP in response to these compounds is not merely an artefact of our fluorescent assay system. In addition, extracellular labelling of cl14 reporter cells using an antibody recognizing the Smo N-terminus confirmed Smo translocation to the plasma membrane in response to stimulation with Hh or compound treatment, another hallmark of Hh pathway activation (Supplementary Fig. S5).

Thus, all three inhibitors selected for the validation experiments activate the Hh signalling cascade, at least down to the level of Smo activation and accumulation at the plasma membrane.

### MAPK pathway inhibitors activate Smo through transcriptional repression of Ptc

The successful validation of these hits encouraged us to investigate the underlying mechanisms of Smo activation. The Hh receptor Ptc is the main, endogenous inhibitor of Smo activation and hence a prime candidate for mediating the observed effects. In cl14 cells and the S2 cells they were derived from, Ptc levels dropped in response to Hh treatment (Fig. 6c,d), presumably reflecting its ligand induced internalization and degradation. However, Ptc is also a target gene of the Hh pathway. Hence, Hh stimulation caused an increase in Ptc levels in the endogenously signalling competent cl8 cells (Fig. 6e). Ptc protein levels decreased in all three tested cell types following treatment with tak-733 or dbn (Fig. 6c–e). This was correlated with a reduction in Ptc transcription in S2 cells (Fig. 6f), which may suffice to explain the greater baseline activation and increased Hh sensitivity of the cells treated with these two inhibitors. Since Ptc downregulation at the protein level was also visible in Cl14 and S2 cells that lack Ci, the effect of the inhibitors must be independent of the Ci mediated regulation of Ptc transcription. Overall, treatment with the two MAPK pathway inhibitors resembles experimental pathway activation by Ptc RNAi, suggesting that Smo or SmoIP are activated by the regular, endogenous machinery.

### The polo inhibitor vlt activates Smo through a distinct pathway that does not activate downstream signal transduction

In contrast, treatment with the polo inhibitor vlt affected neither basal Ptc protein levels (Fig. 6c–e) nor Ptc transcription (Fig. 6f). Treatment of unstimulated cl14 cells with vlt stabilized SmoIP at the protein level comparable to treatment with Hh (Fig. 6c), even though the effect on reporter fluorescence was substantially stronger (Fig. 6a,b). Co-stimulation with vlt and Hh further increased both reporter fluorescence and accumulation of SmoIP (Fig. 6a–c). Consistently, vlt treatment also induced a strong increase in endogenous Smo levels both in signalling dead S2 cells (Fig. 6d) and signaling competent cl8 cells. However, the pronounced cooperativity with Hh seen in vlt treated cl14 and S2 cells (Fig. 6c,d) was absent in cl8 cells (Fig. 6e). Treating signalling competent cl8 cells with Hh upregulated Ptc protein levels, reflecting signal transduction down to the target gene level. In contrast, Ptc levels were reduced rather than increased in vlt treated cl8 cells (Fig. 6e). Thus, vlt appears to influence Smo activation and accumulation through a mechanism that does not activate downstream signalling.

Polo-like kinases contribute to cell cycle regulation by controlling protein ubiquitination and degradation through the anaphase-promoting complex/cyclosome (APC/C)^41^, and have been directly implicated in proteasome activation^42^. We therefore asked whether vlt treatment affected Smo activation by blocking ubiquitination or proteolytic turnover of Smo or the SmoIP sensor. Indeed, multiple proteasome inhibitors present in our library increased sensor fluorescence specifically in non-stimulated cl14 cells, where Smo degradation is expected to occur (Supplementary Fig. S6a). However, vlt treatment had no detectable effect on overall cellular ubiquitination levels, and did also not prevent the expected^12,13^ reduction in SmoIP ubiquitination in response to Hh stimulation (Supplementary Fig. S6b). Moreover, treatment with the proteasome inhibitor MG132 had a pronounced, additive effect with vlt on the fluorescence of non-stimulated cl14 cells. This cooperativity disappeared in the presence of Hh, suggesting that in the non-stimulated cells the excess, stabilized SmoIP protein caused by vlt treatment remained subject to degradation by the proteasome (Supplementary Fig. S6c).

Finally, in unstimulated cells, vlt acted cooperatively the with the phosphatase inhibitor OA but not the PKA activators IBMX and fsk to increase SmoIP fluorescence (Supplementary Fig. S7). Despite the increase in fluorescence, but consistent with the observed lack of downstream signalling activity, the excess SmoIP protein induced by vlt in this respect resembles the inactive SmoIP pool in untreated cl14 cells (Fig. 2).

In summary, the small molecule inhibitors dbn and tak-733 that target Raf and MEK, respectively, appear to downregulate Ptc expression in a Ci independent manner, thereby activating Smo through the endogenous signalling machinery. In contrast, the polo inhibitor vlt promotes Smo activation and accumulation via a different mechanism that by itself fails to trigger downstream signal transduction. While our present experiments cannot dissect these mechanisms in detail, our results clearly demonstrate that the assay is in principle able to uncover Smo activation independent of the underlying molecular mechanism, and is thus fit for purpose.

## Discussion

We have presented a novel, FACS based HTS system in *Drosophila* S2 cells that relies on visualizing Smo activation with the help of a GFP-based sensor. Fluorescence of the sensor is triggered by the phosphorylation induced conformational change in the C-terminal tail during activation of *Drosophila* Smo^15^, although cellular fluorescence levels also reflect the activation-associated stabilization of the sensor protein that is an intrinsic feature of the pathway. Using this system we could follow the Smo response to different Hh concentrations quantitatively and on the single cell level. Furthermore, by screening a library of small molecule inhibitors, we identified groups of compounds with shared molecular targets that affected Smo activation. Our approach thus provides a valuable, additional tool that can in the future be used for the systematic and quantitative investigation of upstream events during Hh pathway activation.

Monitoring the degree of Smo activation for every cell in a large population provides a quantitative resolution that cannot be obtained by bulk, biochemical methods, transcriptional reporters, or imaging based approaches restricted to smaller sample sizes. Pathway response to increasing ligand concentrations is a key problem in developmental biology, where Hh morphogen gradients are interpreted to drive differential target gene expression. According to one model, increasing Hh stimulation increases the number phosphorylated residues in each Smo molecule^21^, presumably by sequential recruitment of distinct serine clusters^43^. Bulk assays such as immunoblots with phospho-specific Smo antibodies indeed suggested such a graded Smo phosphorylation response^43^. However, our experiments revealed that this quantitative response is apparently not due to gradually increasing Smo activation levels in each cell. Instead, submaximal Hh level only induced Smo activation in a subset of all potential target cells. Higher Hh levels increased the ratio of responding to non-responding cells, but did not increase absolute fluorescence levels in the responders. Thus, even in our clonally derived population, individual cells must have different thresholds for Smo activation. Importantly, all cells in the population were, in principle, capable of responding to the Hh upon stimulation at saturating Hh concentrations. It will be interesting to see whether this variation in sensitivity, both between cells or potentially even within one cell over time, also occurs in tissues *in vivo*, which would fit well with recent observations in developmental biology that challenge the notion of graded responses to morphogens. For example, during Sonic Hedgehog mediated dorsoventral pattern formation in the vertebrate spinal cord, different neuronal populations are not generated in precise spatial domains corresponding to well defined pathway activation thresholds, but are instead specified in a noisy pattern that is subsequently resolved by cell sorting^44^. This was, at least partially, attributed to cell and tissue movements causing fluctuations in the local Shh concentrations experienced by the cells. Our observations suggest that differences between individual cells in the sensitivity of their Hh signal transduction machineries may contribute to a noisy pathway activation pattern even at uniform ligand concentrations. It is tempting to speculate that such intrinsic “noise” may even improve the robustness of the patterning process, e.g. by ensuring that the entire range of cellular responses will be realized within a cell population, even if the extremes of the morphogen gradient are missing due to stochastic fluctuations.

A second, interesting observation concerns the activation of Smo by PKA. Depending on the presence or absence of Hedgehog, Smo forms biochemically detectable complexes with PKA^31^ or phosphatases^33^. Expression of constitutively active murine PKA *in vivo* is by itself sufficient to induce fluorescence of the SmoIP reporter and Hh target gene expression^15^, but activation of the endogenous PKA pool by pharmacologically increasing cAMP levels through fsk or IBMX treatment is, at least *in vitro*, incapable of activating Smo. However, both components strongly increase Smo activation in the presence of Hh, suggesting that activated, endogenous PKA can under these conditions act on the Smo sensor. In contrast, the phosphatase inhibitor OA does not require cooperative action of Hh, suggesting that its target phosphatases PP1 or PP2A access Smo in unstimulated cells. These observations recall a constraint we identifed during mathematical simulation of Smo activation^15^, whereby PKA activity must be restricted to the plasma membrane, where it preferentially acts on Smo trafficked there following addition of ligand. Indeed, models in which kinase activity was allowed to act on both the intracellular and membrane associated Smo polls failed to recapitulate the observed system response to Hh stimulation^15^.

In addition to providing quantiative data from single experiments, our assay is scaleable to high throughput mode, up to 384 well plates. We performed a proof of principle screen using a library of small molecules with known molecular targets. Grouping compounds by these targets revealed several compound clusters whose members consistently affected Smo activation. The emergence of these clusters by itself provides a strong validation for the screening results, even though we are not able to extrapolate from the available information on the individual compounds to specific targets in the fly system. Although it was beyond the scope of this study to identify the primary targets of these hits we were able to define a putative mechanism of action for two of the selected compounds: Inhibition of the MAPK cascade by dbn (Raf) and tak-733 (MEK) downregulated Ptc transcription, thus presumably promoting Smo activation by derepression of the endogenous signalling machinery. Since this process was biochemically also confirmed in S2 cells, it appears to be independent of Ci, thus posing an interesting question for future research into the regulation of basal Ptc transcription. Importantly, the effect of the MAPK inhibitors is not due to a general cross-inhibition of cellular kinases: While our inhibitor library did not contain compounds targetting PKA, casein kinase 1/2, or Gprk2, we had directly tested the small molecule PKA inhibitor h89^30^ during the validation phase of our assay. In contrast to MAPK pathway inhibition, inactivation of PKA, as expected, completely abolished Smo reporter fluorescence in response to Hh (Fig. 2a,b).

Treatment of our reporter cells with vlt, a presumptive polo inhibitor, induced Smo activation, accumulation, and relocalisation to the plasma membrane independent of Ptc inhibition. However, this was not associated with signal transduction downstream of Smo, even in the fully Hh sensitive cl8 cells^42^. The notion that Smo remained in a non signalling competent pool, even though sensor fluorescence indicated activation, was also supported by a cooperative effect of vlt treatment with Hh or the phosphatase inhibitor OA, but not the PKA activators fsk or IMBX which, as discussed above, require some prior activation of Smo. Phosphorylation by polo-like kinases, the presumptive targets of vlt, has been implicated in regulation of the APC/C ubiquitination machinery and the proteasome^41,42^. Ubiquitination and degradation also play an important role in regulating Smo. However, we were unable to detect an effect of vlt on either process. To elucidate the mode of Smo activation by vlt we will in the future have to also test possible effects on other posttranscriptional modifications such as sumoylation^14^. Importantly, neither mode of Smo activation could have been uncovered by a screen using a transcriptional readout, which in the case of the Hh pathway are usually based on the Ptc promotor, highlighting the benefits of using multiple, complementary approaches.

Taken together, our experimental results validated the SmoIP assay as a powerful tool for investigating Smo activation that can be employed in high- throughput analysis and yields quantitative data for the investigation of individual cell responses.

### Availability of materials and data

All data generated or analysed during this study are included in this published article and its Supplementary Information files. Cell lines are available from the authors upon request, and will in addition be submitted to appropriate repositories.

## Electronic supplementary material

Supplementary Information

Supplementary table 1

Supplementary table 2
