# Supplementary material for: A cell based, high throughput assay for quantitative analysis of Hedgehog pathway activation using a Smoothened activation sensor
Source: Sci Rep. 2017 Oct 30;7:14341. doi: 10.1038/s41598-017-14767-1 (PMC5662767; doi:10.1038/s41598-017-14767-1)
Supplement: Supplementary file 1 — Supplementary Information [file 41598_2017_14767_MOESM1_ESM.pdf]

A cell based, high throughput assay for quantitative analysis of Hedgehog pathway activation using a Smoothened activation sensor

Evgenii A. Albert, Christian Bökel*

[1]Center for Regenerative Therapies Dresden, Technical University Dresden, Fetscherstr. 105, 01307 Dresden, Germany

* please address correspondence to christian.boekel@tu-dresden.de

Supplementary information:

- Supplementary figures S1 - S7
- Supplementary table legends

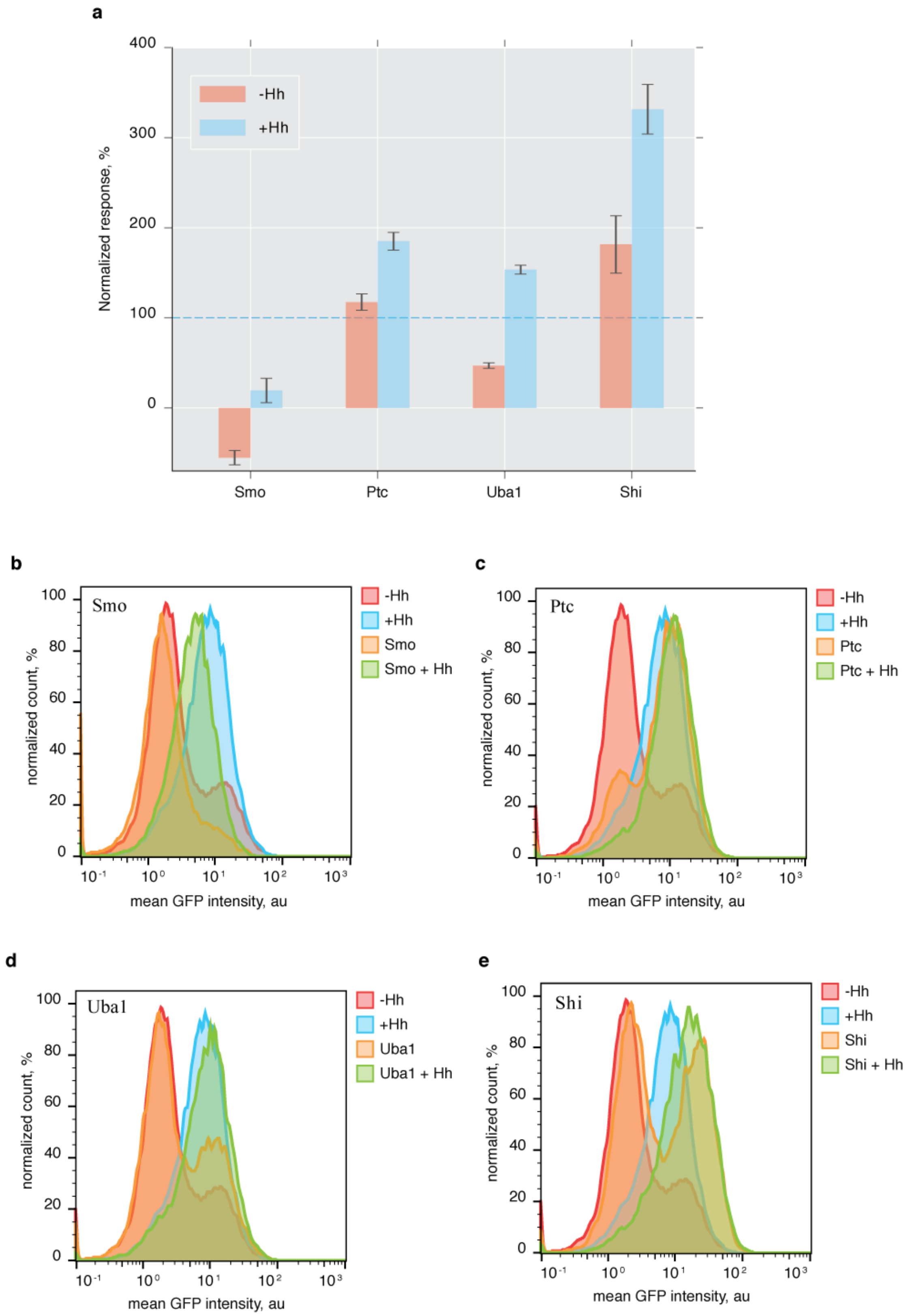
a
400
300
200
100
0
Normalized response, %
-Hh
+Hh
Smo
Ptc
Uba1
Shi
b
Smo
100
80
60
40
20
0
normalized count, %
-Hh
+Hh
Smo
Smo + Hh
10^{-1} 10^{0} 10^{1} 10^{2} 10^{3}
mean GFP intensity, au
c
Ptc
normalized count, %
-Hh
+Hh
Ptc
Ptc + Hh
mean GFP intensity, au
d
Uba1
normalized count, %
-Hh
+Hh
Uba1
Uba1 + Hh
mean GFP intensity, au
e
Shi
normalized count, %
-Hh
+Hh
Shi
Shi + Hh
mean GFP intensity, au

Figure S1. The SmoIP assay captures the endogenous pathway response to RNAi knockdown of Hh pathway components. (**a**) Average effect of RNAi knockdown of Smoothened (Smo), Patched (Ptc), Uba1, and Shibire (Shi) on baseline and Hh induced SmoIP fluorescence response. Effect of Hh stimulation on untreated cells set as 100% (dashed line). N = 3-4 replicates per experiment, mean ± sd. (**b**-**f**) FACS histograms for individual experiments for the indicated RNAi knockdowns.

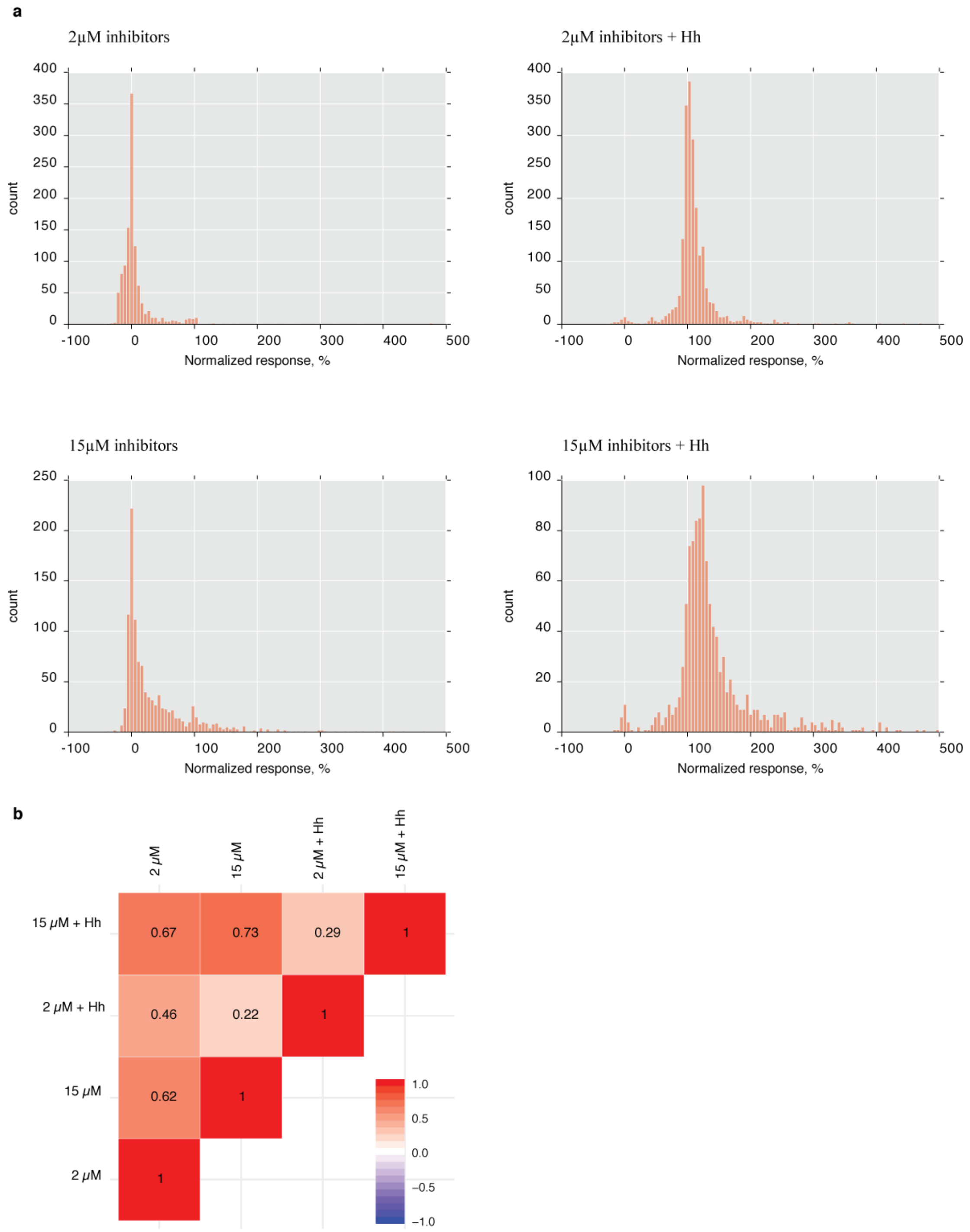

Figure S2. Effect of individual small molecule inhibitors on SmoIP fluorescence. (**a**) Compound number plotted against normalized SmoIP response for the unstimulated and Hh treated cells at the indicated inhibitor concentrations. Unstimulated, untreated cells set to 0%; untreated, Hh stimulated cells set to 100%. (**b**) Pearson correlation between the experiments.

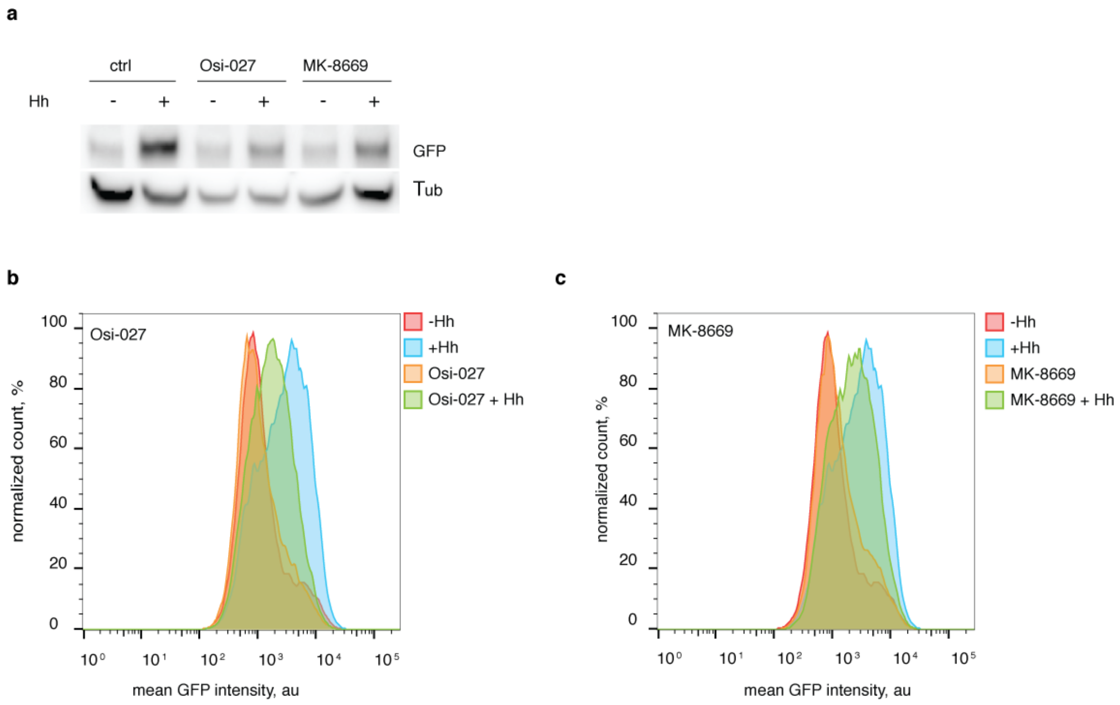

Figure S3. mTOR inhibitors downregulate SmoIP protein level as well as the fluorescent signal. (**a**) SmoIP levels in Cl14 cells treated with mTOR inhibitors for 24h in presence or absence of Hh. (**b-c**) SmoIP fluorescence data measured by FACS corresponding to western blots in (**a**).

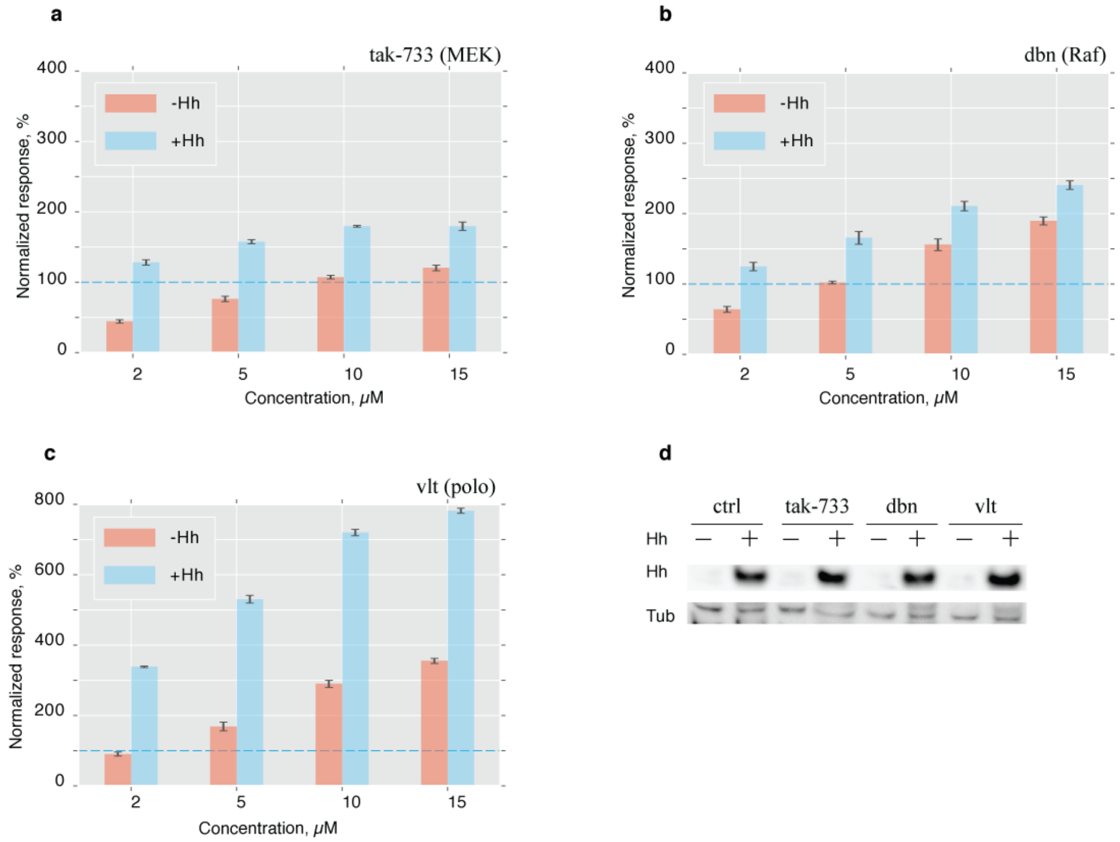

Figure S4. Validation experiments for small molecule inhibitor screen. (**a**-**c**) Dose dependence of small molecule modulation of Smo activation. Normalized SmoIP fluorescence response of cl14 cells treated with the indicated concentrations of tak-733 (**a**), dbn (**b**), or vlt (**c**) in the presence or absence of Hh. N = 4, replicates per experiment, mean ± sd, effect of Hh stimulation on untreated cells set as 100% (dashed line). (**d**) Following inhibitor treatment, extraneously added but no internally produced Hh can be detected by Western blot.

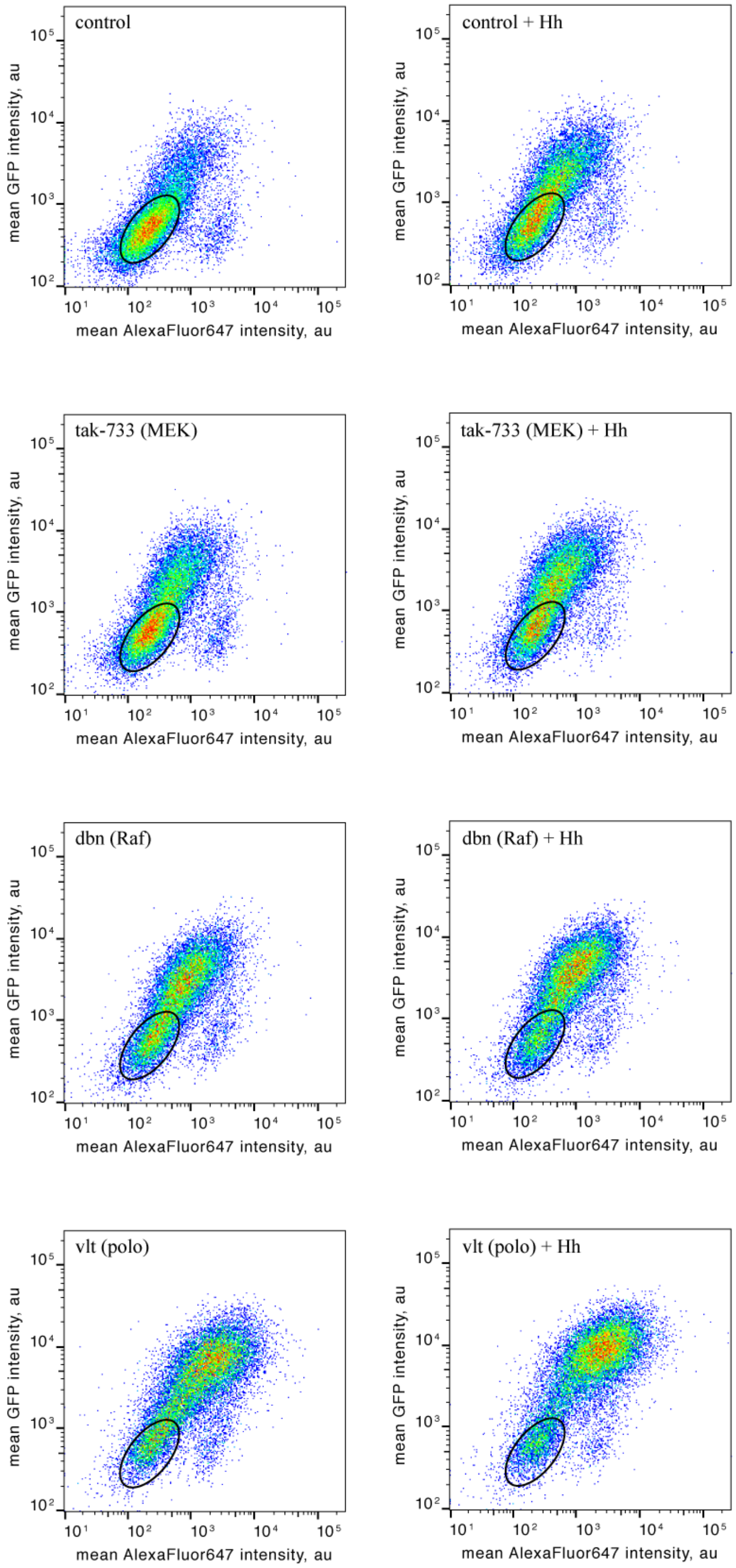
control
control + Hh
tak-733 (MEK)
tak-733 (MEK) + Hh
dbn (Raf)
dbn (Raf) + Hh
vlt (polo)
vlt (polo) + Hh
mean GFP intensity, au
mean AlexaFluor647 intensity, au
10¹ 10² 10³ 10⁴ 10⁵

Figure S5. FACS based quantification of Smo plasma membrane translocation. Cl14 cells were surface stained without permeabilization using a Smo antibody directed against the extracellular, N-terminal domain and a secondary antibody labeled with Alexa-647. Surface localization of Smo for the indicated inhibitor treatments in the presence or absence of Hh was detected by FACS.

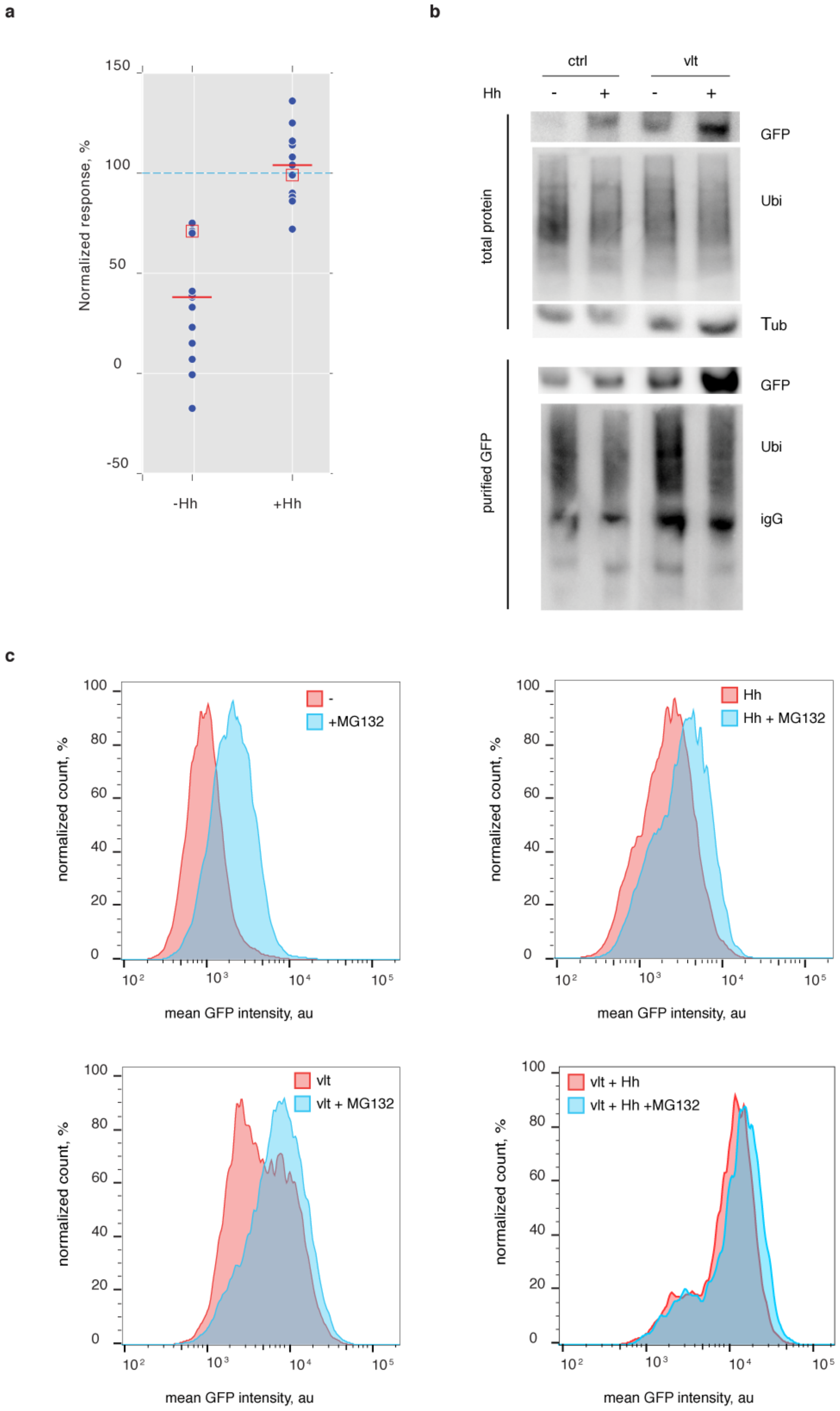
a
b
c
Normalized response, %
150
100
50
0
-50
-Hh
+Hh
ctrl
vlt
Hh
-
+
-
+
total protein
purified GFP
GFP
Ubi
Tub
GFP
Ubi
igG
normalized count, %
mean GFP intensity, au
-
+MG132
Hh
Hh + MG132
vlt
vlt + MG132
vlt + Hh
vlt + Hh +MG132

Figure S6. Vlt and ubiquitination of SmoIP. (**a**) Effect of individual proteasome inhibitors (15μM) on SmoIP fluorescence in untreated and Hh stimulated cells. Red bars indicate median, boxes marks effect of MG132. (**b**). Ubiquitination level of SmoIP after vlt treatment. Upper panel, total protein lysate, lower panel, SmoIP purified from total lysate. Cells were treated with 50μM of MG132 5h before lysis (**c**) FACS quantification of for MG132 effect on SmoIP fluorescence in Hh stimulated and untreated cells corresponding to experiments in (a) and (**b**).

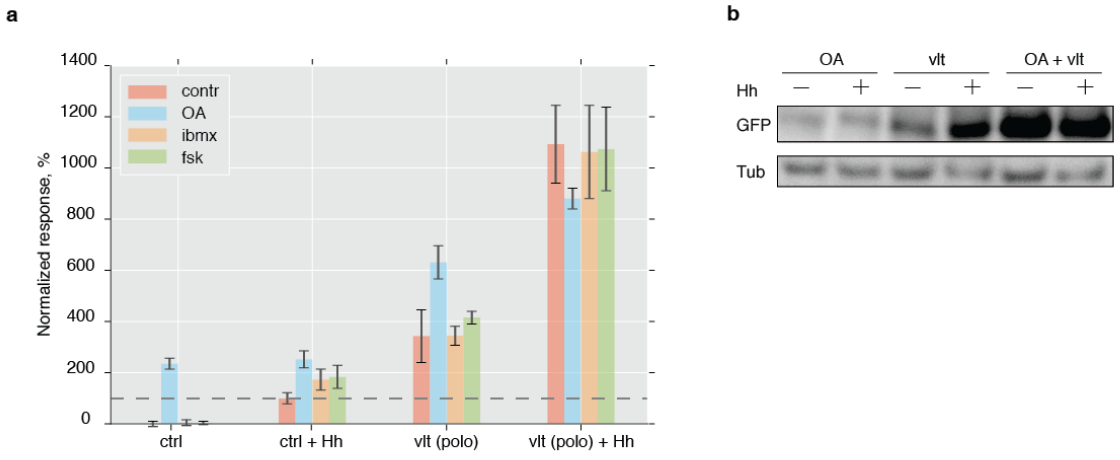

Figure S7. Combined effect of Vlt and PKA modulators. (**a**) Normalized fluorescence response to inhibitor treatment in the presence or absence of the indicated inhibitor combinations. Effect of Hh stimulation of untreated cells set as 100% (dashed line), N = 3, mean ± sd. (**b**) Stabilization of SmoIP as detected by GFP Western blot following OA, vlt, or combined treatment in the presence or absence of Hh.

Supplementary table legends:

Supplementary table 1. Contains raw results of the screen with the following information for each compound: Main target, percent of singlets from total event number, median intensity of GFP channel, normalized GFP intensity (see Methods section), link to the compound description at Selleckchem web site.

Supplementary table 2. Contains raw results of the screen for compounds clustered according to their primary target, with the following information for each cluster: Median GFP intensity for each given cluster under all four screening conditions, number of compounds in the group, primary target of the group.
